# Supplementary material for: A benchmark study of simulation methods for single-cell RNA sequencing data
Source: Nat Commun. 2021 Nov 25;12:6911. doi: 10.1038/s41467-021-27130-w (PMC8617278; doi:10.1038/s41467-021-27130-w)
Supplement: Supplementary file 1 — Supplementary information. [file 41467_2021_27130_MOESM1_ESM.pdf]

Supplementary Figures

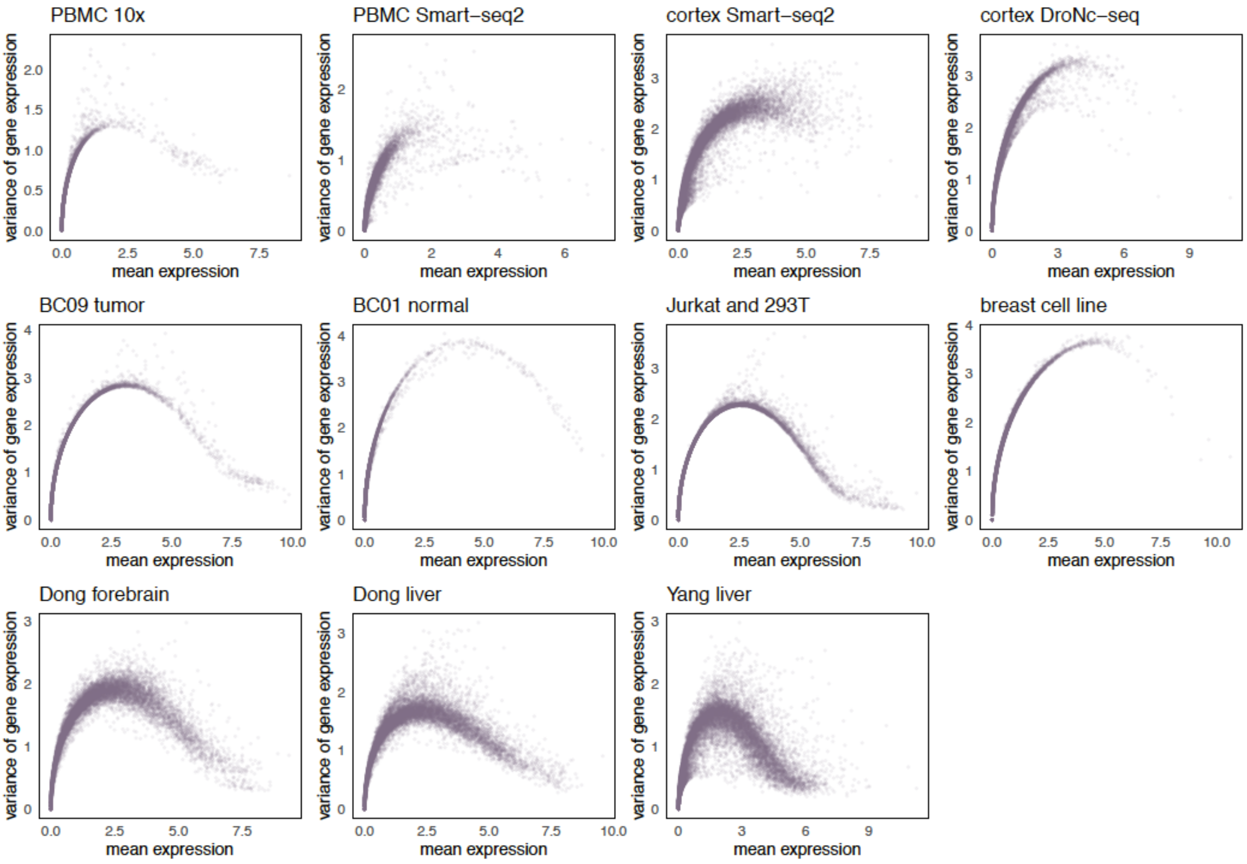

Supplementary Figure 1. Mean-variance relationship of genes across multiple datasets.

We show the property of mean-variance relationship of genes in 11 datasets as an example to demonstrate the variability of data properties across datasets. Top panel shows four datasets of different protocols, the first two from human PBMC samples and the latter two from mouse cortex samples. Middle panel shows two datasets from tissue source and two datasets with cell line source. Bottom panel shows datasets of multiple cell types in mouse sample.

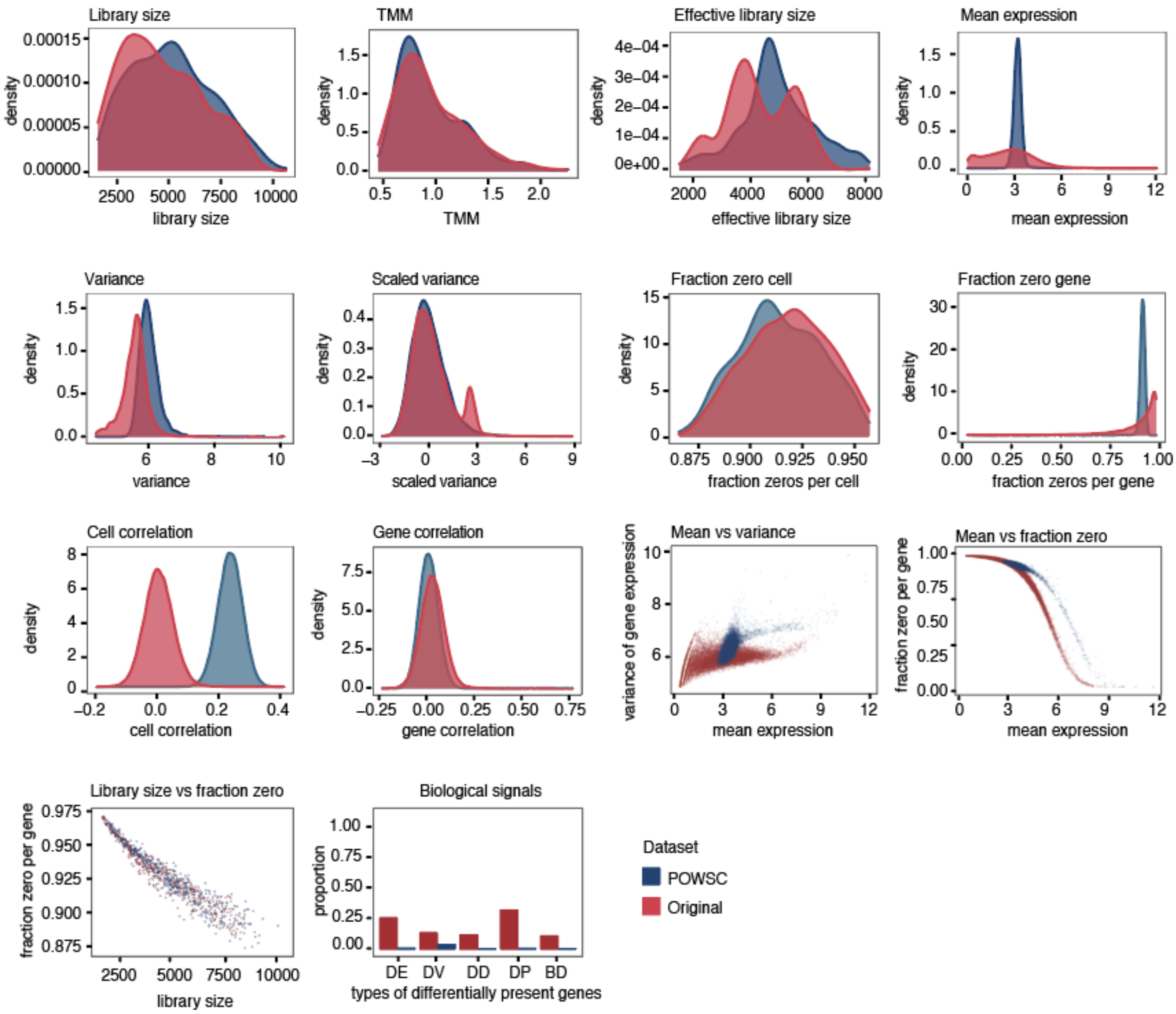

Supplementary Figure 2. Visual representation of the evaluation criteria in properties estimation and biological signals.

As an illustrative example, we compared the simulation data generated by POWSC and the original dataset Soumillon that was used as the reference input. In properties estimation, we compared the concordance of the data characteristics across multiple properties using the KDE statistic. In biological signals, we compared the concordance of the amount of proportion of biological signals in simulated and in real data.

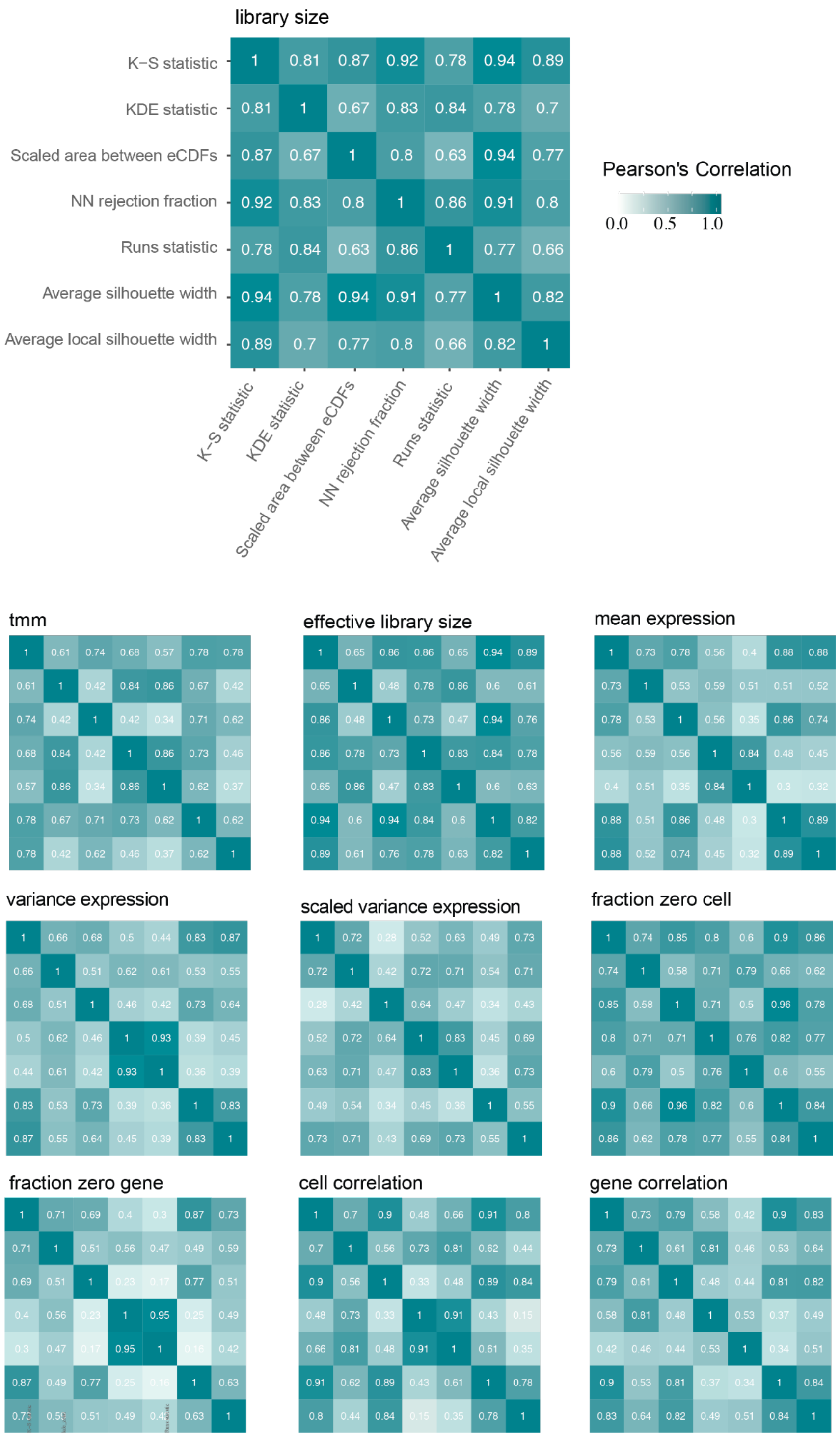

Supplementary Figure 3. Correlation between seven measures on quantifying similarities for univariate properties.

Top panel shows the correlation matrix for the property library size, enlarged for readability of axis labels. Bottom panel shows correlation matrix for the remaining univariate properties. The axis labels are consistent and are not shown for readability of the matrix.

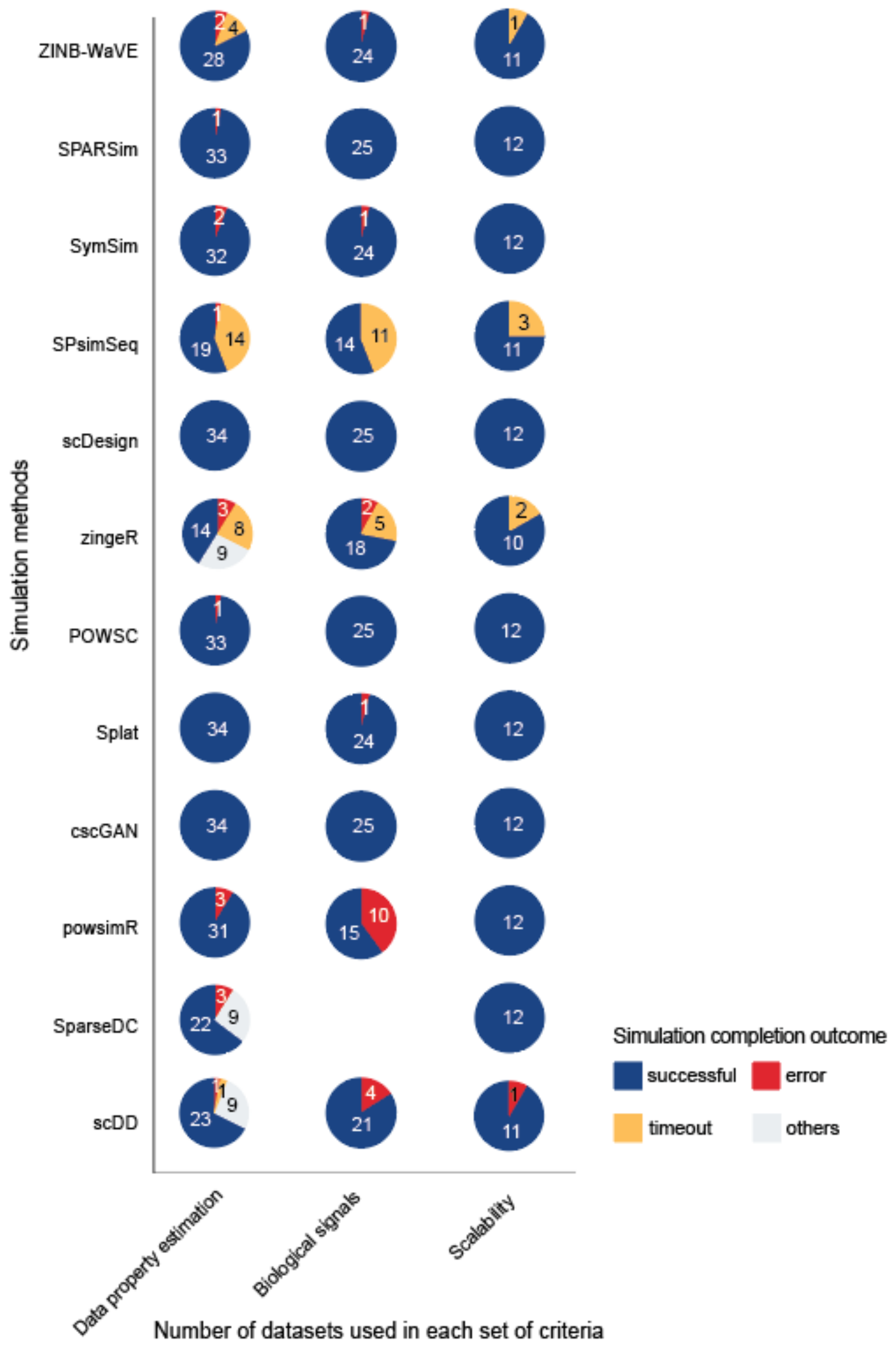

Supplementary Figure 4. Number of datasets used for evaluation and simulation completion outcome

A total of 34 datasets were used for evaluating the simulation methods on data property estimation, 25 datasets were used for evaluating biological signals and 12 datasets were used for evaluating scalability (see Methods). Pie chart denotes the completion outcome of each simulation method. “Successful” indicates the method produced a simulation dataset

within the given time limit. “Error” indicates the method encountered issues during simulation. “Timeout” indicates the method was not able to finish the simulation within the given time limit (see Methods). “Others” indicates a special situation where the methods require the dataset to have two or more cell types and therefore could not be tested on datasets containing a single cell type.

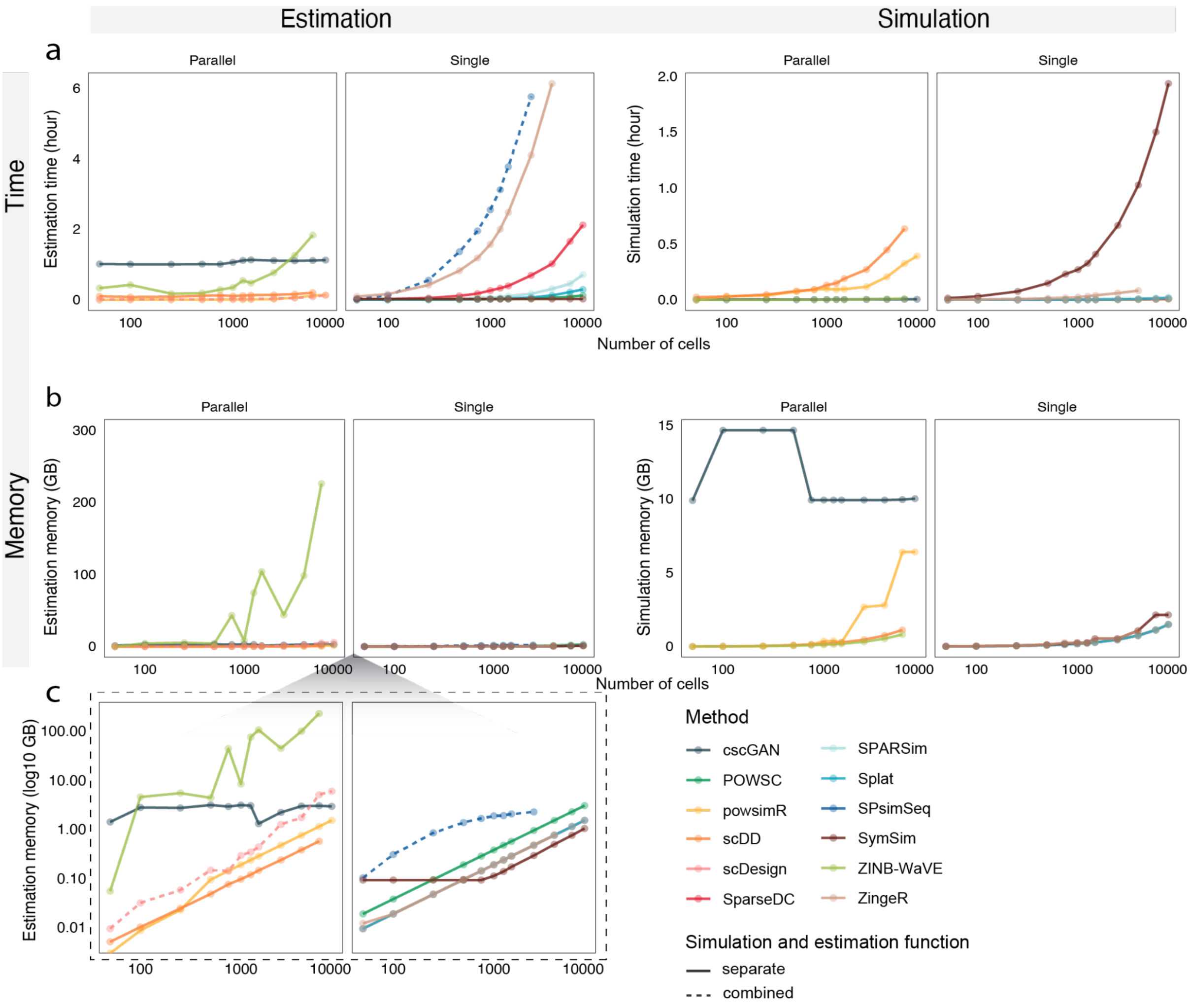

Supplementary Figure 5. Run time and memory consumption of each method.

a Runtime of each method. **b** Maximal memory usage of each method. The number of cells is shown in log10 scale. Methods that support parallel computing and those that only support single core are shown separately. Most methods involve a two-step process of properties estimation and dataset simulation. For those methods, we recorded and shown results for

the two steps separately under the estimation and simulation panels. A solid line was used to indicate these methods. For methods that perform the two steps together in a single function, we displayed the results under the estimation panel. A dashed line was used to indicate these methods. **c** This shows the same result as in **b**, but with the y-axis in log10 scale for enhanced readability.

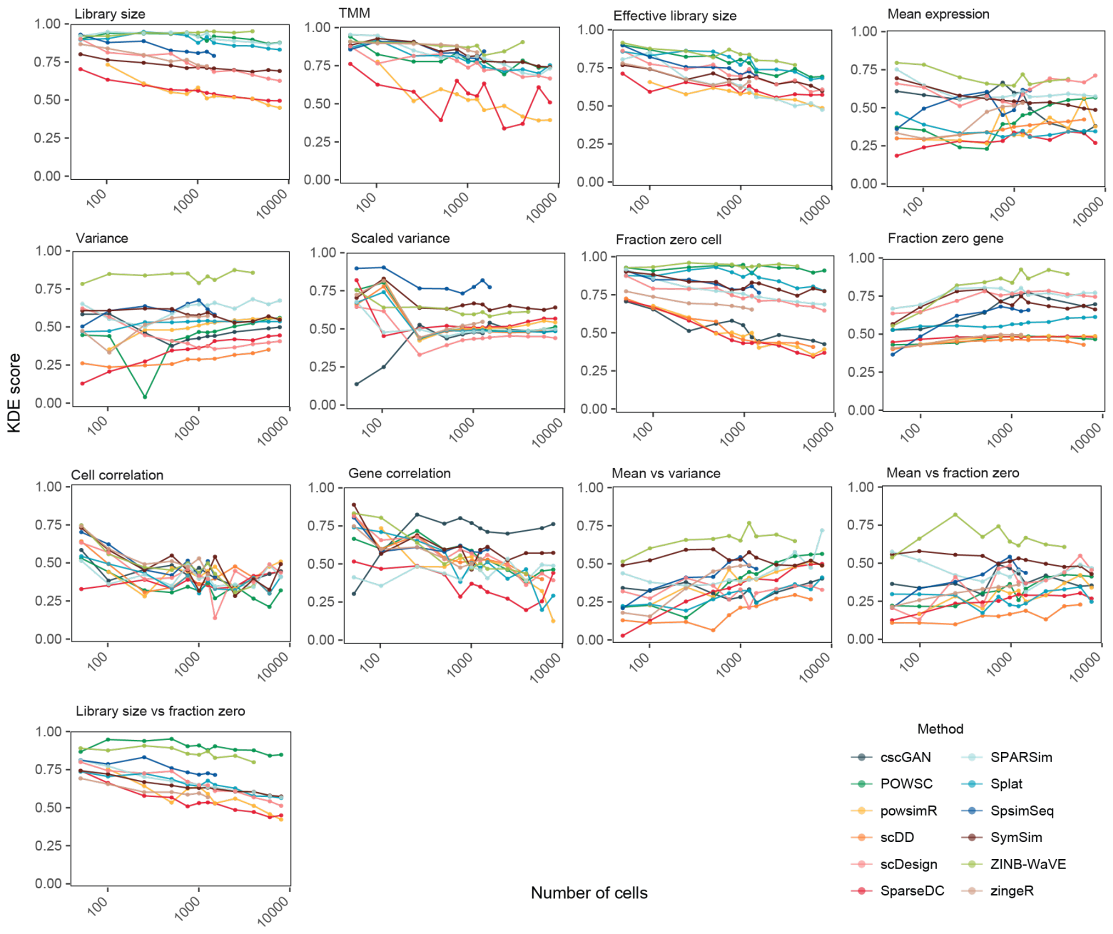

Supplementary Figure 6. Impact of the number of cells on property estimation.

The x-axis shows the number of cells in log10 scale and y-axis shows the score. The line shows the trends with increasing cell numbers. The dot indicates where a measurement is taken. Each measurement was taken three times and the average was shown in the figure.

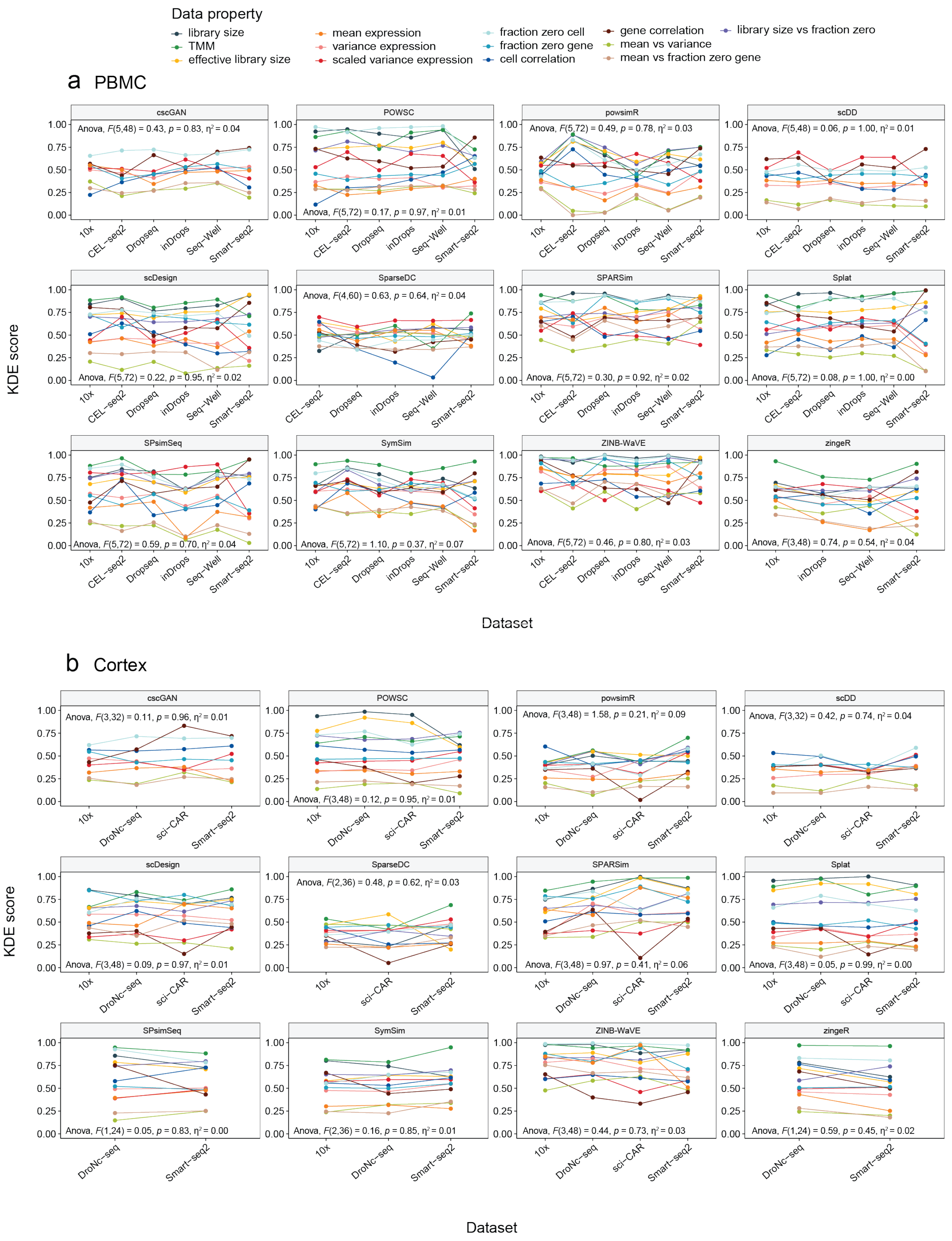

Supplementary Figure 7. Impact of sequencing protocols on data property estimation.

The impact of sequencing protocols on data property estimation using **a** human PBMC data collections and **b** mouse cortex data collections. ANOVA was performed to examine the statistical significance of the change in KDE score due to sequencing protocol effect. The test statistics, effect sizes, degrees of freedom and P-values are shown on each panel.

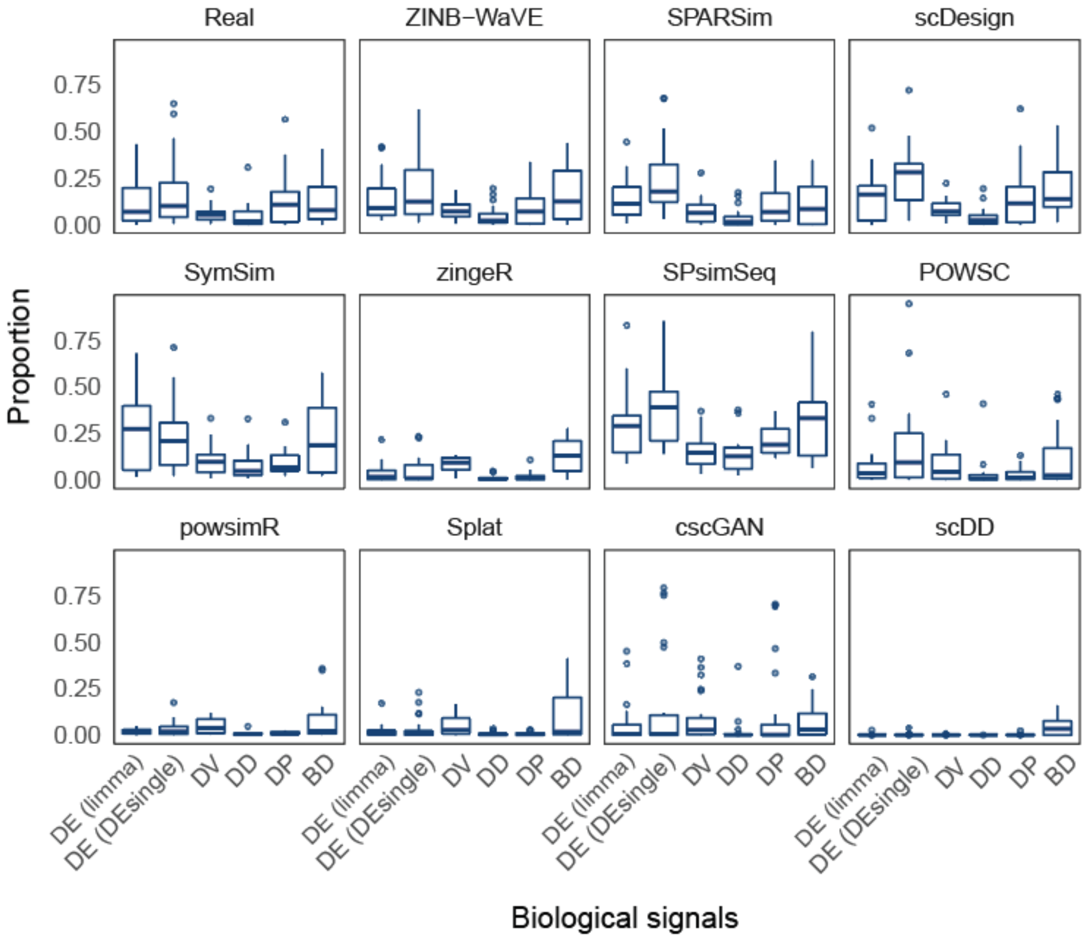

Supplementary Figure 8. Proportion of biological signals in real and simulated data generated by simulation methods.

The boxplots show the distribution of the proportion of biological signals for all datasets examined (*n* = 25 for SPARSim, scDesign, POWSC and cscGAN, *n* = 24 for ZINB-WaVE, SymSim and Splat, *n* = 21 for scDD, *n* = 18 for zinger, *n* = 15 for powsimR, *n* = 14 for SPsimSeq). The proportion of biological signals in the simulated data ideally should be similar to that of the real data. The box represents quartiles, the line represents the median, the lower and upper whisker represents the bottom 25% and top 25% of the data. Outliers are shown as individual data points.

Supplementary Tables

Supplementary Table 1. Additional details of the scRNA-seq simulation methods evaluated in this study.

Methods	Implementation language	Year of publication	Reference (doi)	Software version	Input data (raw/ normalised)	Output data (raw/ normalised)
scDD	R	2016	10.1186/s13059-016-1077-y	1.12.0 (implemented in Splatter)	raw	normalised
Splat	R	2017	10.1186/s13059-017-1305-0	1.12.0	raw	raw
powsimR	R	2017	10.1093/bioinformatics/btx435	1.2.3	raw	raw
SparseDC	R	2017	10.1093/nar/gkx1113	0.1.17 (implemented in Splatter)	raw	raw
zingeR	R	2018	10.1186/s13059-018-1406-4	0.1.0	raw	raw
ZINB-WaVE	R	2018	10.1038/s41467-017-02554-5	1.10.0 (implemented in Splatter)	raw	raw
SymSim	R	2019	10.1038/s41467-019-10500-w	0.0.0.9000	raw	raw
scDesign	R	2019	10.1093/bioinformatics/btz321	1.0.0	raw	raw
SPARSim	R	2020	10.1093/bioinformatics/btz752	0.9.5	both raw and normalised	raw
SPsimSeq	R	2020	10.1093/bioinformatics/btaa105	0.99.13	raw	raw
POWSC	R	2020	10.1093/bioinformatics/btaa607	0.1.0	raw	raw
cscGAN	Python	2020	10.1038/s41467-019-14018-z	GitHub version 379ff6e	raw	normalised

Supplementary Table 2. Detailed simulation strategy of each method.
(*) We used the procedure described in "Evaluation of biological signals" of the Methods section to calculate the proportion of differential expressed genes between the two largest cell types in the real data. This proportion was then used as the input parameter in the simulation function to control the proportion generated in the simulation data.

Methods	Simulation Strategy for evaluating data property estimation	Simulation Strategy for evaluating biological signals
Splat	Estimated the parameters and simulated each cell type separately.	Estimated parameters from the largest cell type in a dataset, set the number of groups to 2 and the proportion of differential expressed (DE) genes to the proportion between the two largest cell types in the dataset (*). This is because the genes in the simulated data do not have a one-to-one matching relationship with the input data and hence it is not possible to combine two simulated data generated from two cell types separately.
powsimR	This method generates DE genes from a homogenous population, for example, a particular cell type from one patient to create two artificial populations. We therefore estimated the parameters and simulated each cell type separately. The proportion of DE and log fold change were set to be a null scenario to maintain the biological signals in the original cell type population.	This method generates DE genes from a homogenous population. We therefore estimated the parameters and simulated the largest cell type. The proportion of DE was set to the proportion between the two largest cell types in the dataset.
SymSim	Estimated the parameters and simulated each cell type separately.	Estimated the parameters and simulated the two largest cell types separately.
scDesign	Estimated the parameters and simulated each cell type separately.	This method generates DE genes from a homogenous population. We therefore estimated the parameters and simulated the largest cell type. The proportion of DE was set to the proportion between the two largest cell types in the dataset
SPARSim	Estimated the parameters and simulated each cell type separately.	Estimated the parameters and simulated the two largest cell types separately. This is because the method returns gene names in the simulated data and therefore we can combine the two datasets and evaluate the biological signals between the two cell types.
SPsimSeq	Estimated the parameters and simulated each cell type separately.	Estimated the parameters and simulated the two largest cell types separately.
POWSC	Estimated the parameters and simulated each cell type separately.	Estimated the parameters and simulated the two largest cell types separately.
zingeR	We estimated and simulated every two cell types at a time with the proportion of DE gene set to 10%. This is the setting used by the authors of this method when comparing their simulated dataset to the original dataset.	We estimated and simulated the two largest cell types at a time with the proportion of DE gene set to the proportion between these two cell types.
scDD	We estimated and simulated every two cell types at a time with the proportion of DE genes set to 10%. This is because the method requires two cell types to be	We estimated and simulated the largest two cell types with the proportion of DE genes set to the proportion between these two cell types.

	simulated at once with a given proportion of DE genes between them.	
ZINB-WaVE	This method takes cell types label into consideration in the parameter estimation step, thus estimation and simulation were performed directly on the entire dataset with cell type labels provided.	Estimation and simulation were performed directly on the entire dataset with cell type labels provided. We then evaluated the biological signals between the two largest cell types.
SparseDC	This method requires two conditions such as treatment and control, with multiple cell types in each condition, as an internal clustering step is performed to differentiate the cell types. We followed the procedure in the SparseDC documentation and split half of the cell types into condition 1 and half of the cell types into condition 2, and specified the number of clusters to be the number of cell types in condition 1 and 2.	Due to the unique setting, we did not evaluate this method for biological signals.
cscGAN	This method takes cell types label into consideration in the parameter estimation step, thus estimation and simulation were performed directly on the entire dataset with cell type labels provided.	Estimation and simulation were performed directly on the entire dataset with cell type labels provided. We then evaluated the biological signals between the two largest cell types.
