# Supplementary material for: A benchmark study of simulation methods for single-cell RNA sequencing data
Source: Nat Commun. 2021 Nov 25;12:6911. doi: 10.1038/s41467-021-27130-w (PMC8617278; doi:10.1038/s41467-021-27130-w)
Supplement: Supplementary file 3 — Description of Additional Supplementary Files. [file 41467_2021_27130_MOESM3_ESM.pdf]

Description of Additional Supplementary Files

File Name: Supplementary Data 1
Description: Details of the datasets used in this study.
